# No advantage of fundoplication in paraesophageal hernia repair: a retrospective multicenter study

**DOI:** 10.1093/dote/doaf036

**Published:** 2025-05-18

**Authors:** Lene Østerballe, Eirik K Aahlin, Rasmus Goll, Mahdi Alamili, Per-Even Storli, Mads V Gran, Cecilie B Lassen, Palle B Miliam, Kim E Mortensen

**Affiliations:** Department of Gastrointestinal Surgery, University Hospital of Northern Norway, St. Olavsgate 70, 9405 Harstad, Norway; Institute of Clinical Medicine, UiT the Arctic University of Norway, Hansine Hansensvei 18, 9019 Tromsø, Norway; Institute of Clinical Medicine, UiT the Arctic University of Norway, Hansine Hansensvei 18, 9019 Tromsø, Norway; Department of Gastrointestinal Surgery, University Hospital of Northern Norway, Hansine Hansensvej 67, 9019 Tromsø, Norway; Institute of Clinical Medicine, UiT the Arctic University of Norway, Hansine Hansensvei 18, 9019 Tromsø, Norway; Department of Gastroenterology, Division of Internal Medicine, University Hospital of Northern Norway, Hansine Hansensvei 67, 9019 Tromsø, Norway; Department of Gastrointestinal Surgery, Hvidovre Hospital, Kettegårdsalle 36, 2650 Hvidovre, Copenhagen, Denmark; Department of Gastrointestinal Surgery, St. Olavs Hospital, Trondheims University Hospital, Prinsesse Kristinas Gate 3, 7030 Trondheim, Norway; MiDT, Nasjonalt forskningssenter for minnimalt invasivt og billedassistert diagnostikk og behandling, St Olavs Hospital, Trondheims University Hospital, Prinsesse Kristinas Gate 3, 7030 Trondheim, Norway; Department of Gastrointestinal Surgery, St. Olavs Hospital, Trondheims University Hospital, Prinsesse Kristinas Gate 3, 7030 Trondheim, Norway; Department of Gastrointestinal Surgery, Hvidovre Hospital, Kettegårdsalle 36, 2650 Hvidovre, Copenhagen, Denmark; Department of Gastrointestinal Surgery, Hvidovre Hospital, Kettegårdsalle 36, 2650 Hvidovre, Copenhagen, Denmark; Institute of Clinical Medicine, UiT the Arctic University of Norway, Hansine Hansensvei 18, 9019 Tromsø, Norway; Department of Gastrointestinal Surgery, University Hospital of Northern Norway, Hansine Hansensvej 67, 9019 Tromsø, Norway

**Keywords:** paraesophageal hernia, nissen fundoplication, foregut surgery, large hiatal hernia, anti-reflux surgery

## Abstract

Paraesophageal hernia repair often includes both gastropexy and fundoplication. The fundoplication may cause persistent side effects, and the necessity of the procedure is uncertain. This study aimed to compare gastropexy with or without fundoplication. A retrospective multicenter study was conducted from three Scandinavian hospitals. Patients, with grade III-IV hiatal hernia, who had a laparoscopic repair with total hernia sack removal, closure of hiatus, gastropexy either with or without Nissen fundoplication were included. Outcomes were per- and postoperative complications, postoperative symptom control, and recurrence. A total of 320 patient cases were included in the study (72 patients with Nissen fundoplication and 248 patients without fundoplication). Baseline variables were comparable between the two groups. We found no difference in perioperative or postoperative events, reflux control or recurrence. Median operation time differed with 49 minutes (*P* < 0.001) in patients with fundoplication (Median: 108 minutes, interquartile range (IQR): 88–131 minutes) compared to patients without fundoplication (59 minutes, IQR = 46–78 minutes). We also found an increased risk for second repair in the fundoplication group (OR 4.3, 95% CI 1.4–13.3). This study shows no benefits of adding a Nissen fundoplication procedure to anterior gastropexy for paraesophageal hernia repair. It was not superior compared to gastropexy alone in terms of postoperative reflux control or preventing recurrence. In contrast, the fundoplication was associated with a four-fold increase of second repair, but the study design limits firm conclusions on this matter.

## INTRODUCTION

Paraesophageal hernia (PEH, grade II-IV hiatal hernias) accounts for ˂5% of hiatal hernias.^1^ Unlike the smaller grade I hiatal hernias, PEH can lead to severe symptoms such as dysphagia, vomiting, weight loss, anemia, reduced pulmonary capacity and potentially emergency situations with incarceration.^2^^,^^3^ The population affected by PEH is primarily elderly individuals with age-related ailments, frailty, and reduced functional status. Surgical intervention is the only curative treatment.

For many years, the procedure has been performed laparoscopically, associated with low mortality and morbidity rates. The surgical approach involves the removal of the hernia sack, the repositioning of the esophagus well below the hiatus, and a hiatoplasty. The debate whether to include a gastropexy and/or a fundoplication has persisted for at least 30 years. Due to insufficient evidence, the latest guidelines do not provide clear recommendations, leaving the decision to each foregut center.^4^

The gastropexy is performed to prevent reherniation.^5–7^ It is considered a low-risk procedure, and it is easy and quick to perform.

The purpose of the anti-reflux procedure is to address any existing and/or postoperative reflux and to prevent recurrence of the hernia.^8–10^ Studies indicate that adding an anti-reflux procedure reduces postoperative reflux and esophagitis^11–16^ but with the risk of dysphagia, chest pain and a complex recurrence if the fundoplication migrates up in the mediastinum.

Many centers now do the paraesophageal repair with some sort of gastropexy (either anteriorly to the interior abdominal wall, to the hiatus or the diaphragm) and an anti-reflux procedure, the fundoplication.^17^ But the clinical significance of the fundoplication, is unclear, as it has not demonstrated a significant impact on overall health-related quality of life or postoperative recurrence.^12^^,^^18^ Other reports suggest that patients operated for PEH can have adequate control of reflux symptoms on proton pump inhibitors (PPIs) after surgery without the necessity of an anti-reflux procedure.^19^^,^^20^

Finally, considering the vulnerability of these patients due to age and comorbidities, this might also explain why some surgeons choose less invasive surgical approaches without anti-reflux procedure when severe reflux is not present.^20–22^

As the issue of fundoplication is not resolved, this study aims to compare gastropexy with or without fundoplication for paraesophageal hernia repair by evaluating peroperative events, postoperative complications, persistent and recurrent postoperative symptoms, radiological recurrence and need of second surgery.

## METHODS

Three hundred ninety-five patient records from three surgical centers in Scandinavia; University Hospital of North Norway (UNN), St. Olav’s Hospital (SOH) in Norway, and Hvidovre Hospital (HH) in Denmark were retrospectively analyzed. All centers were highly specialized upper gastrointestinal centers treating adult patients (>18 years) with paraesophageal hernia from their respective catchment areas. Records from all operated patients in the period from November 01, 2018 to October 31, 2023 were examined. UNN lacked a standardized surgical protocol for patients with paraesophageal hernia prior to 2022, which accounts for the low patient number from this area. However, first author introduced the surgical method for paraesophageal hernias from HH to UNN in 2022, drawing on her previous work at HH. A paraesophageal hernia was defined as hiatal hernia grade II-IV.^23^ Exclusion criteria were (1) previous surgery of the esophagus or stomach, including earlier hiatal hernia surgery; (2) simultaneous surgery for other gastrointestinal diseases, including obesity surgery; (3) patients not having a gastropexy as part of the procedure for paraesophageal hernia; (4) emergency PEH repair because of strangulated necrotic stomach with the need of resection.

This study was accepted as a quality assurance project by the ethics committee and data protection officer in Denmark (F-23057940) and Norway (ref. 03222 and 662,003, respectively).

Research design and data analysis was built according to the guidelines from http://www.equator-network.org.

### Study outcomes

Our primary outcomes were perioperative events, postoperative complications and secondary outcomes included long-term symptom control and recurrence of the hiatal hernia.

### Data collection

Outcome variables were collected from patient records and stored in a Redcap Database. Baseline information included sex, age, body mass index, Charlson co-morbidity index,^24^ earlier abdominal surgery, symptoms from the paraesophageal hernia, preoperative manometry and pH measurements, grade of paraesophageal hernia based on CT or X-ray/barium swallow and time to surgery (elective, semi-acute). Operative data comprised surgical procedure, duration of operation, use of mesh, and perioperative adverse events like perforation of stomach, esophagus or intestine, lesion of the spleen or other unplanned excessive bleeding, and conversion to laparotomy.

Postoperative data included duration of hospital stay, postoperative complications according to Clavien-dindo score,^25^ Comprehensive complication index,^26^ re-admission within 90 days, and mortality within 30 days. Re-intervention within 30 days was considered a postoperative complication.

Long-term symptom control was assessed by the surgeon in the outpatient clinic as part of a postoperative follow-up program after 3 months at HH and UNN. SOH did not consistently follow-up the patients, but they were referred back for new examinations in case of persistent or new symptoms. Consequently, the follow-up period also involved re-referral of the study patients due to recurrent symptoms. Patients with persistent or recurrent symptoms underwent a CT or X-ray/barium swallow to rule out recurrence of the hiatal hernia. Time of follow up was defined from day of operation to either (1) radiological verified recurrence, (2) death, (3) end of study period 28-02-2024.

### Surgical procedure

All patients were operated according to SAGES guidelines of treatment of paraesophageal hernia.^4^ All patients received preoperative antibiotic prophylaxis. All procedures were either performed or supervised by subspecialized upper gastrointestinal surgeons. Laparoscopy was standard procedure. The herniated stomach and hernial sac were retracted from the mediastinum. In case of a fundoplication procedure the sac was excised, otherwise it was left in place beneath the hiatus. The hiatal opening was reduced in size with non-resorbable continuous or interrupted sutures. A relaxing incision was not performed. When using a mesh, it was placed, covering the hiatus as a U-shape, anchored with absorbable sutures to the diaphragm. All meshes used were biological (hiatal Bio-A mesh, Gore or Biodesign Hiatal Hernia Graft, CookMedical). In case of fundoplication, it was performed as a 360° Nissen wrap with three non-absorbable sutures making the fundoplication; two of the sutures included the esophagus. The short gastric vessels were divided to avoid esophageal constriction. Finally, an anterior gastropexy was performed on every patient. In this procedure, the stomach was attached to the anterior abdominal wall. It was done either transcutaneous with 3–4 interrupted non-absorbable sutures or continuously with a non-absorbable suture. The sutures were placed beneath the left costal margin with 2 cm between the interrupted sutures. The continuous technique involved anchoring the upper part of the stomach to the posterior fascia of the abdominal wall over minimum 5 cm.

### Study groups

Patients underwent same preoperative examinations with endoscopy, CT and/or barium swallow X-ray and if main complain were reflux, they underwent a pH-study. The surgical procedure across the involved centers was similar, with the only difference being the fundoplication procedure. The other steps of the operation; removal of the hernia and hernia sack, hiatus plasty, and anterior gastropexy, were similar. Gastropexy without fundoplication for patients without severe reflux, was a standardized procedure at two of the involved hospitals (UNN and HH). The third hospital (SOH) conducted repairs similarly but always incorporates a 360° Nissen fundoplication. Preoperative esophageal manometry was not consistently performed at any of the centers and did not result in any changes to management. At the surgical centers not performing fundoplication for paraesophageal repair per standard, PEH patients primarily complaining of reflux, underwent a pH study before the operation. If this study showed a pathological pH index, fundoplication was considered. The patient cohort consisted entirely of Danish or Norwegian individuals, two countries which are very similar in terms of socio-economic and health status with equal access to a public healthcare system. The indications for surgery, as well as perioperative and postoperative care, including outpatient follow-up, were similar across the centers.

Thus, patients were divided into two groups: Gastropexy with or without fundoplication.

### Statistical analysis

Statistical analysis was performed using the statistical software package SPSS®, version 29. Continuous variables were presented as median (interquartile range). For Gaussian distributed data, parametric tests were used (independent samples student’s t, two-sided) while non-Gaussian distributed data were tested by non-parametric method (Mann–Whitney U). Categorical variables were reported as numbers with percentages and compared using the Chi-squared or Fisher’s exact test as appropriate. Cox regression and Kaplan–Meier analyses were utilized for factors influencing recurrence. A *P*-value <0.05 was considered statistically significant. For Cox regression analysis we checked the assumption of proportional hazards by examining log–log plots in the final model and found no violations.

## RESULTS

### Patients’ characteristics

**Fig. 1:**
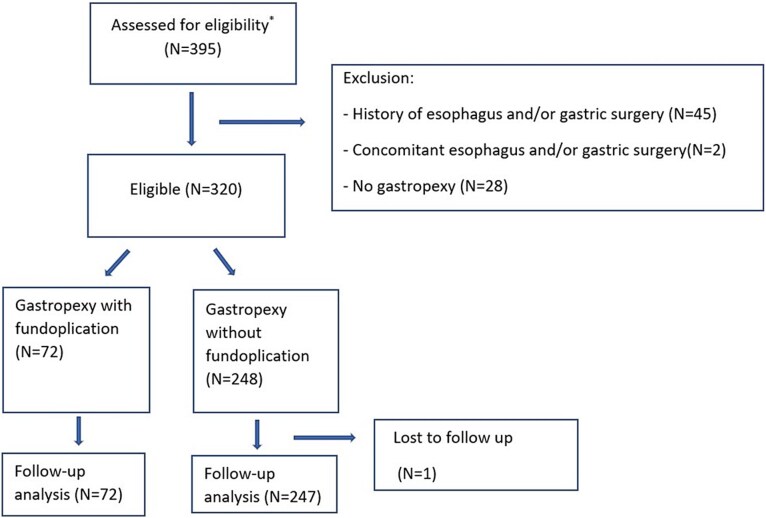
Flow chart of patient selection according to Consort guidelines. ^*^Patients operated for paraesophageal hernia between year 2018 and 2023.

Figure 1 shows the patient selection of all 395 patients operated for paraesophageal hernia during a 5-year period in the three surgical centers. A total of 320 patients were eligible for analysis. Seventy-two patients were operated with gastropexy with fundoplication; 248 patients were operated similarly but without fundoplication. Fourteen patients had a temporary gastrostomy tube inserted as part of the gastropexy. Baseline characteristics are outlined in Table 1. The groups were comparable with no significant difference among the baseline variables (Table 1). Semi-emergent cases were defined as those requiring surgery within 14 days. Depending on severity of symptoms and CT scan results, some semi-emergent cases followed a regimen of endoscopy and gastric tube decompression, restricting oral intake to fluids and eventually combining this with parenteral nutrition for 7–10 days until operation. Median operation time differed with 49 minutes (*P* < 0.001) in patients with fundoplication (108 minutes, IQR 88–131 minutes) compared to patients without fundoplication (59 minutes, IQR 46–78 minutes). More meshes were used in the fundoplication group (*P* = 0.041).

**Table 1:** Patient demographics by gastropexy with or without fundoplication

Gastropexy with fundoplication (N = 72)		Gastropexy without fundoplication (N = 248)	*P* ^a^
Female	59 (82)	185 (75)	0.128
Age (years)	72 (64–76)^b^	73 (65–79)^b^	0.864^c^
Body Mass Index	28 (24–30)^b^	27 (25–30)^b^	0.916^c^
Charlson co-morbidity index ≥3	51 (71)	174 (70)	0.913
PEH grade III	62 (86)	209 (84)	0.853
PEH grade IV	10 (14)	39 (16)	
**Hospital of origin**			
HH	5 (7)	230 (93)	
UNN	0	13 (5)	
SOH	67 (93)	5 (2)	
**Symptoms before operation**			
Nausea	3 (4)	20 (8)	0.196
Dysphagia	43 (60)	92 (37)	<0.001
Vomiting	23 (32)	115 (46)	0.020
Reflux	13 (18)	27 (11)	0.082
Pain	48 (67)	185 (75)	0.120
Dyspnoea	23 (32)	63 (25)	0.170
**Manometry before operation**	9 (13)	23 (9)	0.755
Normal	7 (10)	18 (7)	
Dysfunctional motility	2 (3)	4 (2)	
Achalasia	0	1 (0.4)	
**pH-measurement before operation**	8 (11)	19 (8)	0.399
Normal	4 (6)	13 (5)	
pH Index>4,5	4 (6)	6 (2)	
**Operation**			
Elective setting	55(76)	171 (69)	0.290
Subacute setting <14 days	17 (24)	77 (31)	
Length of operation/minute	108 (88–131)^b^	59 (46–78)^b^	<0.001
Use of mesh	15 (21)	29 (12)	0.041

Values in parentheses are percentages unless indicated otherwise.

HH, Hvidovre Hospital; PEH, Paraesophageal hernia; SOH, St. Olav’s Hospital; UNN, University Hospital of North Norway.

a x2-test or Fischer’s exact test as appropriate.

b Values are median (IQR); PEH, paraesophageal hernia.

c Independent samples t-test.

### Per- and postoperative complications

Table 2 summarizes surgical details and postoperative outcomes. No differences were observed between the groups regarding intraoperative complications, length of hospital stay, re-admission within 30 days or postoperative complications. In a multivariate analysis, the fundoplication was not associated with postoperative complications (OR 1.69, 95%CI: 0.79; 3.62 *P* = 0.17). Four patients died within 30 days after surgery. These four patients where 77 years or older and had severe comorbidity (CCS >3). They were all treated with sub-acute surgery, without fundoplication.

**Table 2:** Peri- and postoperative outcome data

Gastropexy with fundoplication (N = 72)		Gastropexy without fundoplication (N = 248)	*P* ^a^
**Intraoperative events**	4 (6)	5 (2)	0.120
- perforation of esophagus	2		
- perforation of stomach		3	
- perforation of colon		1	
- severe bleeding/lesion of spleen	2	1	
Conversion laparotomy	2 (3)	2 (1)	0.219
**Postoperative complications:** Mild (CD score 1–2)	10 (14)	18 (7)	0.069
Severe (CD score 3–5)	7 (10)	22 (9)	0.490
Comprehensive complication Index	21 (15–32)^b^	21 (9–38)^b^	0.320^c^
Length of hospital stay/days	3 (2–6)^b^	1 (1–7)^b^	0.703^c^
Re-admission <90 days, all causes	8 (11)	34 (14)	0.349
Mortality <30 days	0	4 (2)	0.357

Values in parentheses are percentages unless indicated otherwise.

a x2-test or Fischer’s exact test as appropriate.

b Values are median (IQR); CD-score: Clavien-Dindo score.

c independent samples t-test.

### Long-term symptom control and recurrence

Data on long-term symptom control and recurrence were available in 319 patients. Median follow up time was 24.4 months (IQR: 10.7–42.7 months). Table 3 shows the patients that either had recurrent or persisting symptoms reported at planned follow-up consultation or by re-referral to the out-patient clinic. Dysphagia and chest pain were predominating postoperative symptoms in both groups but were more present in the fundoplication group. Among patients with preoperative dysphagia (N = 135), 10 out of 43 patients (23.3%) still had dysphagia after PEH operation with fundoplication, while nine out of 92 patients (9.8%) who underwent PEH operation without fundoplication had persistent dysphagia postoperatively (*P* = 0.036). In the group without preoperative dysphagia (N = 184), nine out of 146 patients (6.1%) in the group without fundoplication, and two out of 27 patients (7.4%) with fundoplication developed dysphagia after surgery (*P* = 0.820). Reflux did not seem to be a significant problem postoperatively in either of the groups. All patients with persisting or recurrent symptoms received a CT or an X-ray/barium swallow. Fifty patients in total had radiological recurrence. The majority were diagnosed with recurrence within the first year after primary repair. Thirteen out of fifty (26%) of the patients with re-herniation had a grade I hiatal hernia recurrence. Thirty-seven out of fifty (74%) were grade II-IV recurrences. Some patients needed a revisional procedure (7/72 patients in the fundoplication group and 6/247 patients in the group without fundoplication, OR: 4.3, 95% CI:1.4;13.3). All of them were PEH grade III or IV recurrence.

**Table 3:** Long-term symptom control and recurrence

Gastropexy with fundoplication (N = 72)		Gastropexy without fundoplication (N = 247)	*P* ^a^
Persisting/recurrence of symptoms	18 (25)	50 (20)	0.238
**Reported symptoms**—dysphagia	12 (17)	18 (7)	0.036
- vomiting	1 (1)	12 (5)	0.670
- reflux	1 (1)	6 (2)	0.596
- dyspnoea	1 (1)	4 (2)	0.681
- chest pain	12 (17)	23 (9)	0.065
Recurrence of hiatal hernia^b^	13 (18)	37 (15)	0.316
Reoperation because of recurrence	7 (10)	6 (2)	0.010

Values in parentheses are percentages unless indicated otherwise.

a x2-test or Fischer’s exact test as appropriate.

b diagnosed by CT or X-ray/barium swallow.

### Risk factors for recurrence

**Fig. 2:**
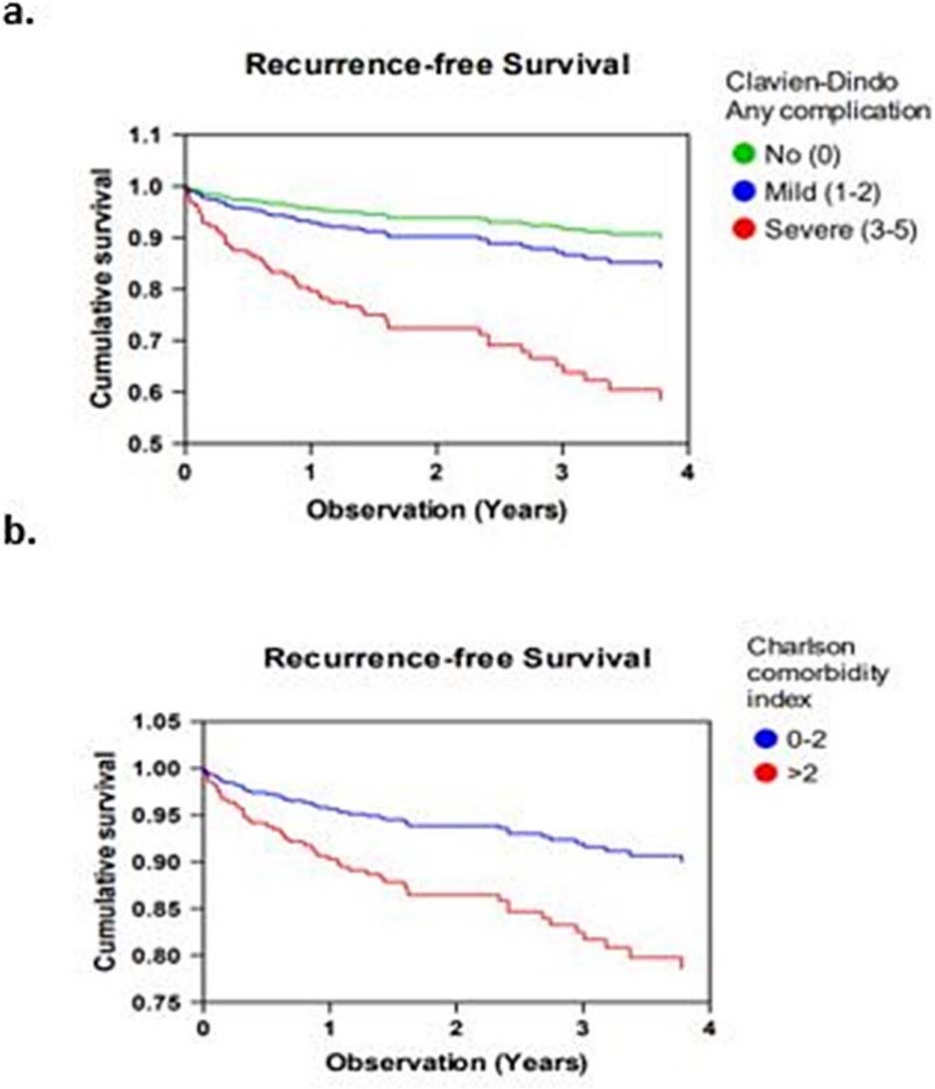
Multivariate Cox proportional hazards for risk factors of recurrence. Cox regression model with potential confounding variables (age, fundoplication, subacute operation, type III or IV paraesophageal hernia, Charlson Comorbidity Index score ≥ 3, postoperative complications, mesh use) entered by backward, conditional method. Variables found to be significant to the risk of hiatal hernia recurrence are (a) Clavien-dindo score ≥ 3 HR 5.6 (2.8–11.3) *P* < 0.001. (b) Charlson co-morbidity index ≥3: HR 2.2 (1.0–5.0) *P* = 0.034.

**Fig. 3:**
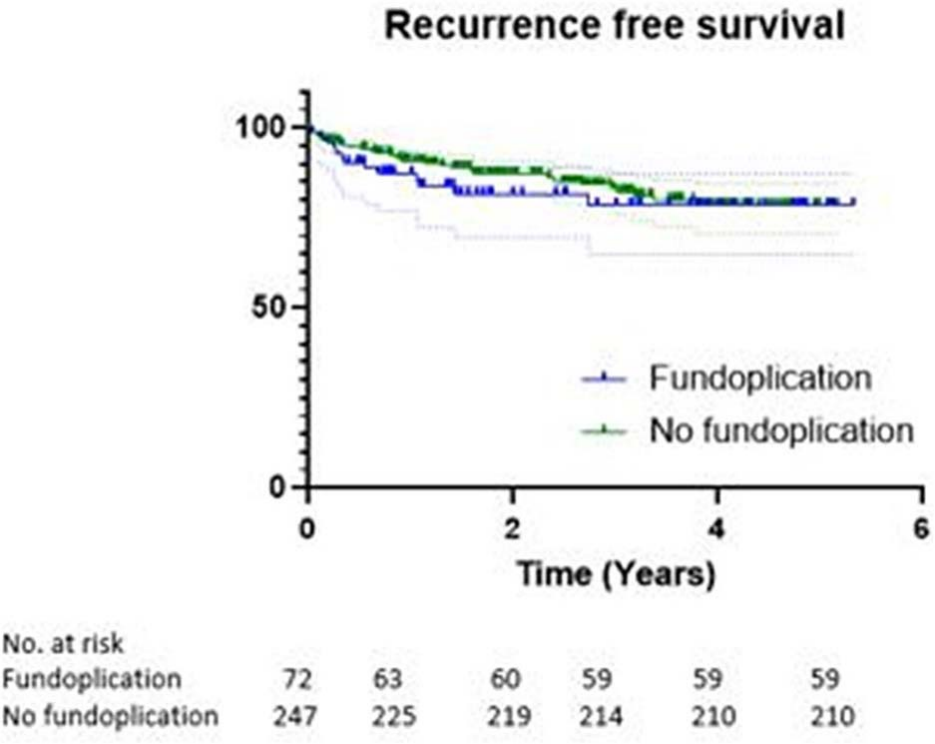
Kaplan Meyer analysis of hiatal hernia recurrence stratified by gastropexy with or without fundoplication.

Uni- (Kaplan Meyer) and multivariate (cox regression) analysis present the risk factors found to be significantly associated with recurrence (Fig. 2 and Fig. 3). We found postoperative severe complications and comorbidity were the only significantly associated risk factors for a hiatal hernia recurrence. The fundoplication procedure did not protect against recurrence (Fig. 3). We tested the Cox regression model for effects of hospital of origin by entering this factor in the final model. The factor was not significant, nor did it change the conclusions of the model.

## DISCUSSION

The findings in this study suggest that a fundoplication is unnecessary in paraesophageal hernia repair. It did not protect against either postoperative reflux or recurrence, which is the main reason for performing the fundoplication procedure. There are not many studies investigating anterior gastropexy without fundoplication for paraesophageal hernia repair; and those that exist are case studies involving a small number of patients who are often fragile and have comorbid conditions.^19^^,^^21^^,^^22^^,^^27^ These studies report acceptable outcomes for this patient population. The novel aspect of our study is that the gastropexy without fundoplication procedure is standardized in two of the included centers and not solely offered as a less invasive option for fragile patients. This aligns with two recent studies: a randomized controlled trial comparing anterior gastropexy with no gastropexy for paraesophageal hernia, which showed that only 20% of patients underwent fundoplication, and that this procedure did not prevent recurrence.^7^ Additionally, a retrospective study involving 1155 patients also found no benefit of fundoplication.^28^

The surgical procedure time was nearly doubled (Table 1) when fundoplication was performed. This increase may be attributed not only to the fundoplication procedure itself but also to the necessity of dividing vasa breves and excising the hernia sac to create sufficient space for the fundoplication. Given the pressures faced by public healthcare systems and the ongoing need for efficiency improvements each year, the substantial decrease in operating time for the group without fundoplication is noteworthy.

Since we did not perform a follow-up CT/X-ray on all our patients, but only on those with recurrent or persisting symptoms, we cannot determine the true recurrence rate. However, the symptomatic recurrence rates were similar between the two groups; nevertheless, the necessity for a second repair was significantly higher in the group undergoing fundoplication. This could be ascribed to more severe symptoms arising from a recurrence with fundoplication improperly positioned above the hiatus. It may also relate to local practices and criteria for reoperation in each center. Moreover, the patients in the fundoplication group consistently had a 360° Nissen fundoplication. It is suggested in earlier studies that a partial or anterior wrap; the 270° Toupe or 180° Dor’s fundoplication, might result in less dysphagia.^9^^,^^29^ Preoperative esophageal manometry could have been valuable in these patients. Unfortunately, guidelines are unclear regarding recommendation for manometry study before paraesophageal hernia repair, so it has not been a standard preoperative examination at the included centers.

While reflux is the primary justification for fundoplication, reflux did not appear to be a significant issue postoperatively in the group without fundoplication. However, postoperative reflux was more frequent in this group, although this was not statistically significant and did not lead to an increased rate of secondary repairs. We did not analyse the intake of proton pump inhibitors (PPIs) since our experience suggests that PPIs are prescribed to patients exhibiting a cluster of various symptoms, which may not necessarily correlate with actual reflux. One could argue that a follow-up gastroscopy for grading esophagitis or pH measurement would have been beneficial to objectively assess the reflux issue.^15^ A study evaluating postoperative reflux after myotomy for achalasia and the LYON consensus guidelines on GERD have not found conclusive correlations between symptoms, endoscopic findings, or pH measurements, raising question about the necessity of these investigations as outcome measures for the severity of reflux symptoms.^30^^,^^31^ Furthermore, our study population typically has an average age of ⁓70 years, with a very low risk of developing Barrett’s esophagus or dysplasia postoperatively if not already present preoperatively.

The 30-day mortality rate was four out of 248 in the gastropexy group without fundoplication, compared to 0 out of 72 in the group with fundoplication (*P* = 0.357). This may be a statistical outlier, due to differences in group size or selection practices at the three centers. However, all patients who died within 30 days postoperatively were *a priori* high-risk cases with severe comorbidities having sub-acute procedures and given the right selection we suggest that both procedures are equally safe.

Limitations of this study include its retrospective design, and the absence of standardized patient reported outcome measures (PROMs), relying instead on physician-reported postoperative symptom control. The absence of PROMs introduces affinity and confirmation biases and the inconsistency of patient follow-up among the centers introduces a selection bias. The reliability of our data is limited by the fact that long-term follow-up depends on referrals back to the clinic. However, in Scandinavia, the public healthcare system facilitates a short and direct pathway from primary care to specialist treatment, ensuring that patients with postoperative symptoms are promptly re-referred to the operating center.

Another weakness of our study is the unequal distribution of surgical techniques and volume of procedures among the study centers, with fundoplication almost exclusively being performed at one of the participating centers and a high rate of procedures in another center. While the groups were similar regarding baseline data, the difference in sample size and surgical technique among the hospitals may influence the reliability and interpretation of the findings. However, entering the hospital of origin in our regression analyses showed no effects on our conclusions.

In conclusion, our study did not find any advantage in the 360° fundoplication procedure as part of the paraesophageal hernia repair. In contrast, fundoplication was associated with a four-fold increase in the need for a second repair, without better symptom control or preventing recurrence compared to gastropexy alone. We suggest a pragmatic approach for patients with paraesophageal hernia, indicating that a 360° fundoplication may not be necessary in the absence of reflux. Due to the retrospective design and inherent limitations of our study, we refrain from making definitive conclusions and emphasize the need for future prospective studies.

## Adresses of institutions at which the work was carried out together

Department of Gastrointestinal Surgery, University Hospital of Northern Norway, St Olavs Gate, NO-Harstad, Norway

Department of Gastrointestinal Surgery, Hvidovre Hospital, Kettegårdsalle, DK-Hvidovre Denmark

Department of Gastrointestinal Surgery, St. Olavs Hospital, Trondheims University Hospital, Prinsesse Kristinas Gate, NO-Trondheim, Norway

## References

[1] Hyun J J, Bak Y T. Clinical significance of hiatal hernia. Gut Liver 2011; 5: 267–77. 21927653 10.5009/gnl.2011.5.3.267 PMC3166665

[2] Daigle C R, Funch-Jensen P, Calatayud D, Rask P, Jacobsen B, Grantcharov T P. Laparoscopic repair of paraesophageal hernia with anterior gastropexy: a multicenter study. Surg Endosc 2015; 29: 1856–61. 25294550 10.1007/s00464-014-3877-z

[3] Puri A, Patel N M, Sounderajah V et al. Development of the ParaOesophageal hernia SympTom (POST) tool. Br J Surg 2022; 109: 727–32. 35640625 10.1093/bjs/znac139 PMC10364681

[4] Daly S, Kumar S S, Collings A T et al. SAGES guidelines for the surgical treatment of hiatal hernias. Surg Endosc 2024; 38: 4765–75. 39080063 10.1007/s00464-024-11092-3

[5] Poncet G, Robert M, Roman S, Boulez J C. Laparoscopic repair of large hiatal hernia without prosthetic reinforcement: late results and relevance of anterior gastropexy. J Gastrointest Surg 2010; 14: 1910–6. 20824385 10.1007/s11605-010-1308-6

[6] Ponsky J, Rosen M, Fanning A, Malm J. Anterior gastropexy may reduce the recurrence rate after laparoscopic paraesophageal hernia repair. Surg Endosc 2003; 17: 1036–41. 12658421 10.1007/s00464-002-8765-2

[7] Petro C C, Ellis R C, Maskal S M et al. Anterior Gastropexy for Paraesophageal hernia repair: a randomized clinical trial. JAMA Surg 2024; 160: 247–55. 10.1001/jamasurg.2024.5788 PMC11904718 39714889

[8] Dreifuss N H, Schlottmann F, Molena D. Management of paraesophageal hernia review of clinical studies: timing to surgery, mesh use, fundoplication, gastropexy and other controversies. Dis Esophagus 2020; 33: 1–9. 10.1093/dote/doaa045 PMC8344298 32476002

[9] Analatos A, Lindblad M, Ansorge C, Lundell L, Thorell A, Håkanson B S. Total versus partial posterior fundoplication in the surgical repair of Para-oesophageal hernias: randomized clinical trial. BJS Open 2022; 6: zrac034, 1–9. 10.1093/bjsopen/zrac034 PMC9070466 35511051

[10] Clapp B, Hamdan M, Mandania R et al. Is fundoplication necessary after paraesophageal hernia repair? A meta-analysis and systematic review. Surg Endosc 2022; 36: 6300–11. 35024937 10.1007/s00464-022-09024-0

[11] van der Westhuizen L, Dunphy K M, Knott B, Carbonell A M, Smith D E, Cobb WSt. The need for fundoplication at the time of laparoscopic paraesophageal hernia repair. Am Surg 2013; 79: 572–7. 23711265

[12] Svetanoff W J, Pallati P, Nandipati K, Lee T, Mittal S K. Does the addition of fundoplication to repair the intra-thoracic stomach improve quality of life? Surg Endosc 2016; 30: 4590–7. 26905576 10.1007/s00464-016-4796-y

[13] Solomon D, Bekhor E, Kashtan H. Paraesophageal hernia: to fundoplicate or not? Ann Transl Med 2021; 9: 902. 34164536 10.21037/atm.2020.03.106 PMC8184421

[14] Li Z T, Ji F, Han X W et al. Role of fundoplication in treatment of patients with symptoms of hiatal hernia. Sci Rep 2019; 9: 12544. 31467314 10.1038/s41598-019-48740-x PMC6715856

[15] Furnée E J, Draaisma W A, Gooszen H G, Hazebroek E J, Smout A J, Broeders I A. Tailored or routine addition of an antireflux fundoplication in laparoscopic large hiatal hernia repair: a comparative cohort study. World J Surg 2011; 35: 78–84. 20957361 10.1007/s00268-010-0814-8 PMC3006643

[16] Müller-Stich B P, Achtstätter V, Diener M K et al. Repair of Paraesophageal hiatal hernias—is a fundoplication needed? A randomized controlled pilot trial. J Am Coll Surg 2015; 221: 602–10. 25868406 10.1016/j.jamcollsurg.2015.03.003

[17] Gerdes S, Schoppmann S F, Bonavina L et al. Management of paraesophageal hiatus hernia: recommendations following a European expert Delphi consensus. Surg Endosc 2023; 37: 4555–65. 36849562 10.1007/s00464-023-09933-8 PMC10234895

[18] Furnée E J, Draaisma W A, Simmermacher R K, Stapper G, Broeders I A. Long-term symptomatic outcome and radiologic assessment of laparoscopic hiatal hernia repair. Am J Surg 2010; 199: 695–701. 19892314 10.1016/j.amjsurg.2009.03.008

[19] Morris-Stiff G, Hassn A. Laparoscopic paraoesophageal hernia repair: fundoplication is not usually indicated. Hernia 2008; 12: 299–302. 18214636 10.1007/s10029-008-0332-x

[20] Dara V, Croo A, Peirsman A, Pattyn P. Necessity of fundoplication and mesh in the repair of the different types of paraesophageal hernia. Acta Gastroenterol Belg 2019; 82: 251–6. 31314184

[21] Bruenderman E H, Martin R C G, Kehdy F J. Outcomes after laparoscopic Gastropexy as an alternative for Paraesophageal hernia repair. Jsls 2020; 24: e2020.00059. 10.4293/JSLS.2020.00059 PMC7688338 33293783

[22] Rosenberg J, Jacobsen B, Fischer A. Fast-track giant paraoesophageal hernia repair using a simplified laparoscopic technique. Langenbecks Arch Surg 2006; 391: 38–42. 16391947 10.1007/s00423-005-0008-2

[23] Fuchs K H, Kafetzis I, Hann A, Meining A. Hiatal hernias revisited-a systematic review of definitions, classifications, and applications. Life (Basel) 2024; 14: 1145. 39337928 10.3390/life14091145 PMC11433396

[24] Charlson M E, Pompei P, Ales K L, MacKenzie C R. A new method of classifying prognostic comorbidity in longitudinal studies: development and validation. J Chronic Dis 1987; 40: 373–83. 3558716 10.1016/0021-9681(87)90171-8

[25] Dindo D, Demartines N, Clavien P A. Classification of surgical complications: a new proposal with evaluation in a cohort of 6336 patients and results of a survey. Ann Surg 2004; 240: 205–13. 15273542 10.1097/01.sla.0000133083.54934.ae PMC1360123

[26] Slankamenac K, Graf R, Barkun J, Puhan M A, Clavien P A. The comprehensive complication index: a novel continuous scale to measure surgical morbidity. Ann Surg 2013; 258: 1–7. 23728278 10.1097/SLA.0b013e318296c732

[27] Geha A S, Massad M G, Snow N J, Baue A E. A 32-year experience in 100 patients with giant paraesophageal hernia: the case for abdominal approach and selective antireflux repair. Surgery 2000; 128: 623–30. 11015096 10.1067/msy.2000.108425

[28] Lyons J, Chatha H N, Boutros C et al. Fundoplication at the time of paraesophageal hernia repair does not decrease the rate of hernia recurrence or postoperative reflux. Surg Endosc 2024; 39: 577–81. 39448405 10.1007/s00464-024-11317-5

[29] Trepanier M, Dumitra T, Sorial R et al. Comparison of dor and Nissen fundoplication after laparoscopic paraesophageal hernia repair. Surgery 2019; 166: 540–6. 31416603 10.1016/j.surg.2019.06.031

[30] Ponds F A, Oors J M, Smout A, Bredenoord A J. Reflux symptoms and oesophageal acidification in treated achalasia patients are often not reflux related. Gut 2021; 70: 30–9. 32439713 10.1136/gutjnl-2020-320772 PMC7788183

[31] Gyawali C P, Yadlapati R, Fass R et al. Updates to the modern diagnosis of GERD: Lyon consensus 2.0. Gut 2024; 73: 361–71. 37734911 10.1136/gutjnl-2023-330616 PMC10846564

